# Supplementary material for: A comprehensive analysis of ribonucleotide reductase subunit M2 for carcinogenesis in pan-cancer
Source: PLoS One. 2024 Apr 18;19(4):e0299949. doi: 10.1371/journal.pone.0299949 (PMC11025932; doi:10.1371/journal.pone.0299949)
Supplement: S1 File — (DOCX) [file pone.0299949.s001.docx]

**Supporting Figures**

**
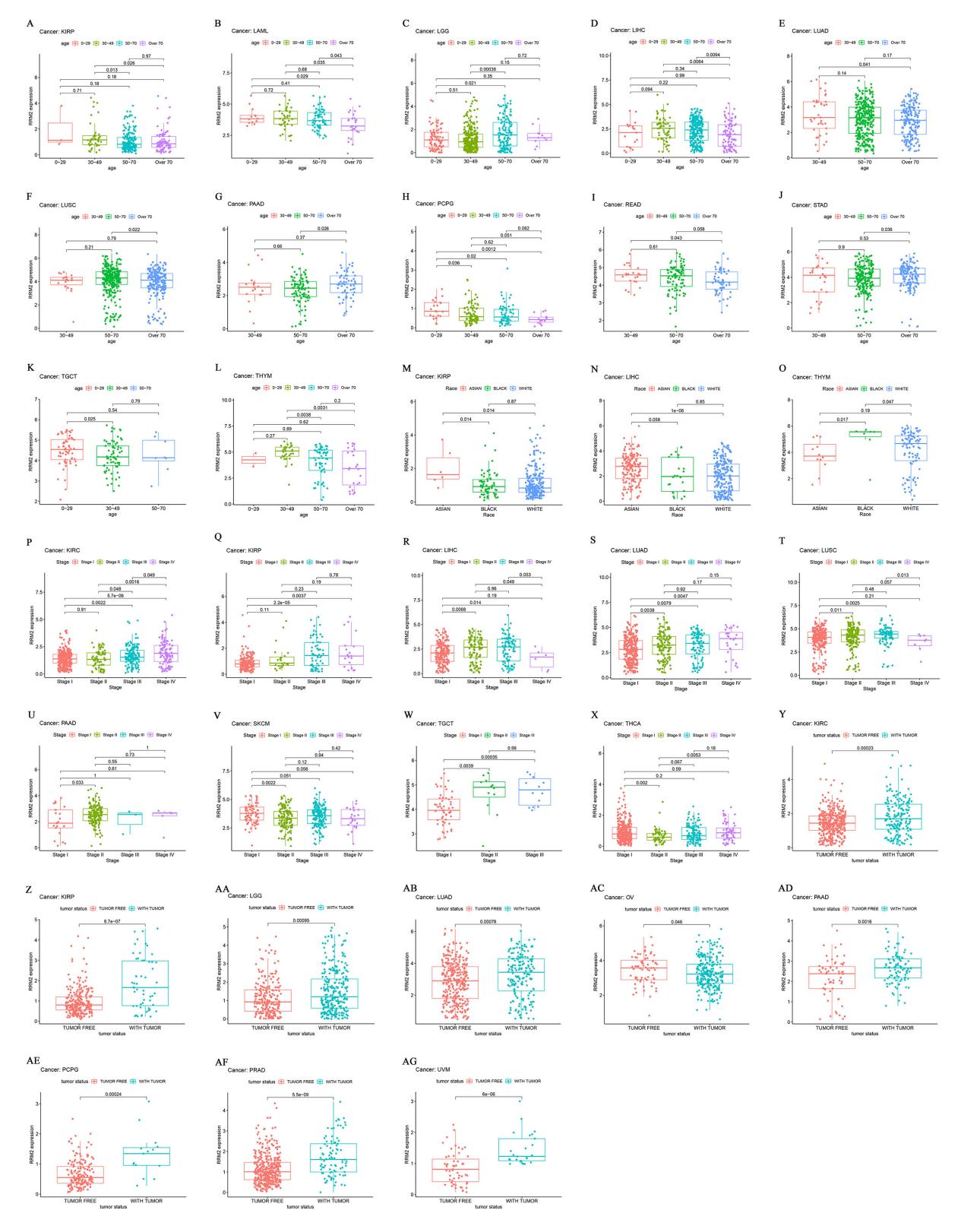
**

**S1 Fig. The expression level of RRM2 and its clinical correlation in pan-cancer.** (A-L) The clinical correlation between RRM2 expression level and age in KIRP, LAML, LGG, LIHC, LUAD, LUSC, PAAD, PCPG, READ, STAD, TGCT and THYM, respectively. (M-O) The clinical correlation between RRM2 expression level and race in KIRP, LIHC and THYM, respectively. (P-X) The clinical correlation between RRM2 expression level and tumor stage of tumor patient in KIRC, KIRP, LIHC, LUAD, LUSC, PAAD, SKCM, TGCT, and YHCA, respectively. (Y-AG) The clinical correlation between RRM2 expression level and tumor status of tumor patient in KIRC, KIRP, LGG, LUAD, OV, PAAD, PCPG, PRAD and UVM, respectively. The number above the horizontal line represents the p-value between the two groups.


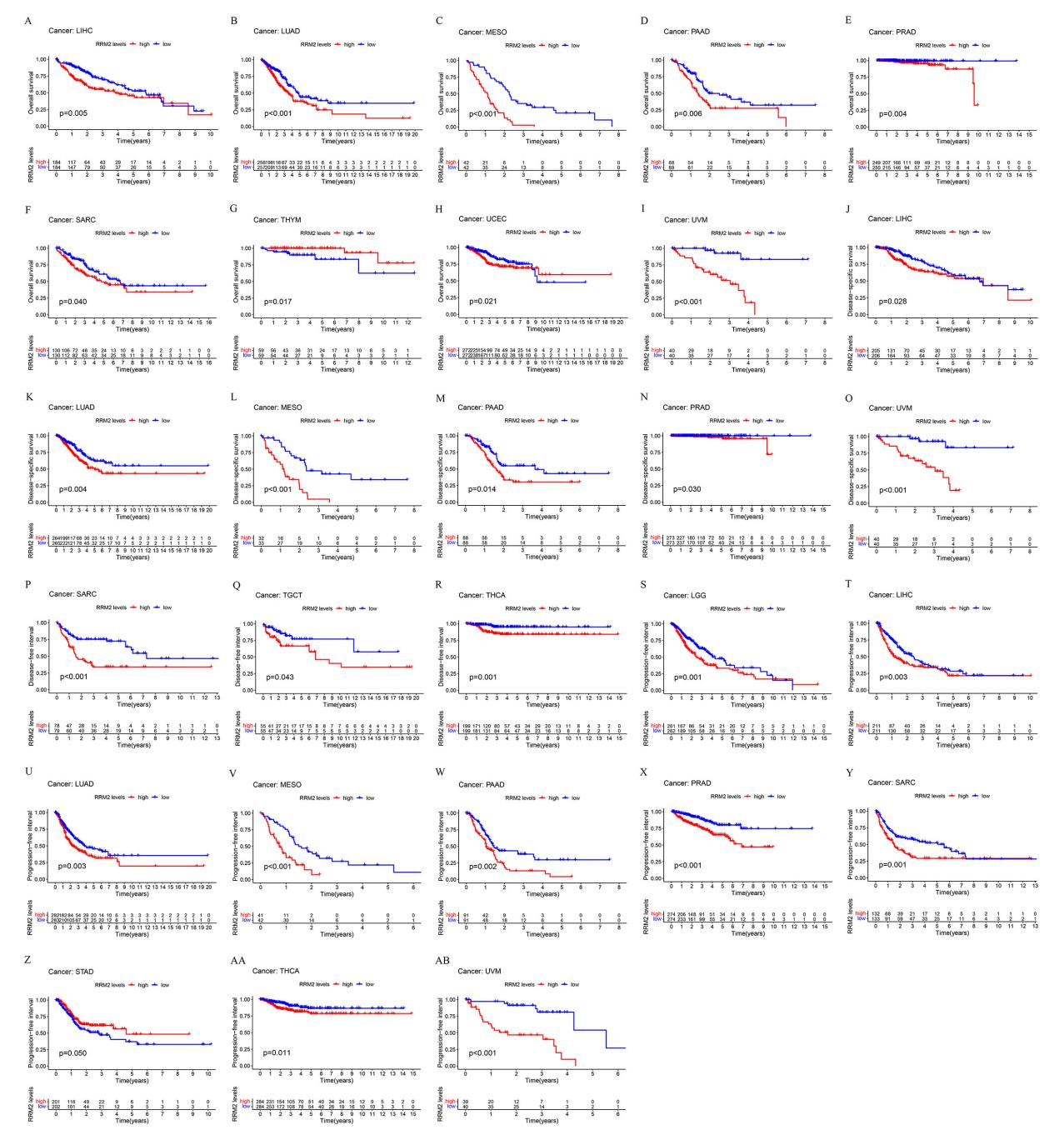


**S2 Fig. Prognostic value of RRM2 in pan-cancer.** (A-AB) Correlation between expression level of RRM2 and OS, DSS, DFI and PFI in pan-cancer, using the Kaplan-Meier method. OS, Overall survival (years); DSS, Disease-specific survival (years); DFI, Disease-free interval (years); PFI, Progression-free interval (years).


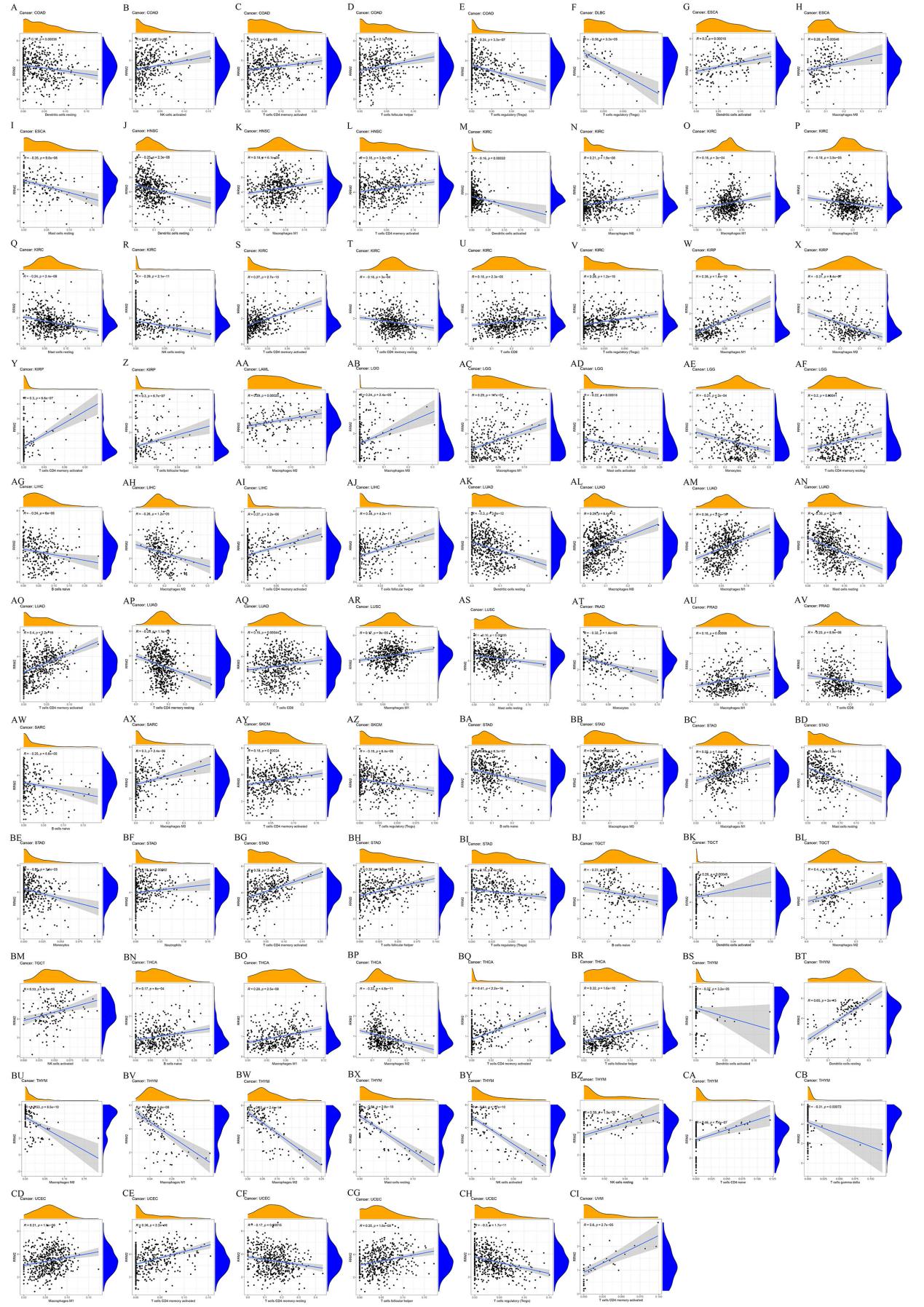


**S3 Fig. Correlation analysis between RRM2 expression and immune infiltration of cancer-associated cells.** (A-CI) The correlation between RRM2 gene expression and the infiltration level of diverse immune cells in pan-cancer.


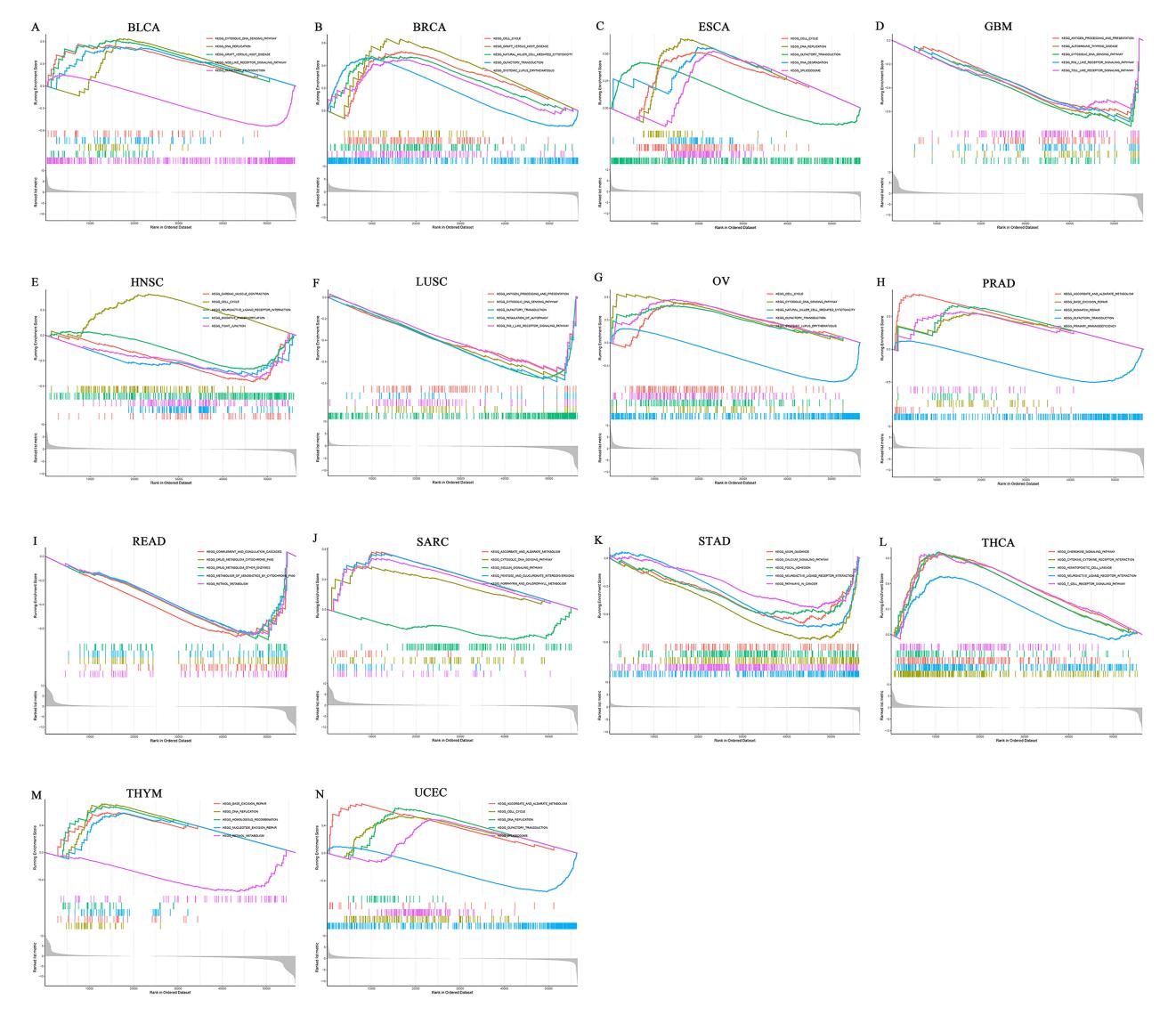


**S4 Fig. Gene set enrichment analysis (GSEA) of RRM2 in pan-cancer.**

1. N) Gene set enrichment analysis (GSEA) of RRM2 in pan-cancer.

**Supporting Tables**

| KEGG ID | Cancer Types | Number of  Cancer Types |
| --- | --- | --- |
| KEGG_CELL_CYCLE | BLCA, BRCA, ESCA, HNSC, OV, STAD, THCA, UCEC | 8 |
| KEGG_OLFACTORY_TRANSDUCTION | BLCA, BRCA, ESCA, LUSC, OV, PRAD, UCEC | 7 |
| KEGG_CYTOSOLIC_DNA_SENSING_PATHWAY | BLCA, ESCA, GBM, LUSC, OV, SARC | 6 |
| KEGG_DNA_REPLICATION | BLCA, BRCA, ESCA, STAD, THYM, UCEC | 6 |
| KEGG_ANTIGEN_PROCESSING_AND_PRESENTATION | BLCA, BRCA, ESCA, GBM, LUSC | 5 |
| KEGG_NATURAL_KILLER_CELL_MEDIATED_CYTOTOXICITY | BLCA, BRCA, ESCA, OV, THCA | 5 |
| KEGG_SPLICEOSOME | BLCA, ESCA, OV, STAD, UCEC | 5 |
| KEGG_ASCORBATE_AND_ALDARATE_METABOLISM | BRCA, PRAD, SARC, UCEC | 4 |
| KEGG_CELL_ADHESION_MOLECULES_CAMS | ESCA, READ, STAD, THCA | 4 |
| KEGG_NEUROACTIVE_LIGAND_RECEPTOR_INTERACTION | ESCA, HNSC, STAD, THCA | 4 |
| KEGG_PENTOSE_AND_GLUCURONATE_INTERCONVERSIONS | BRCA, PRAD, SARC, UCEC | 4 |
| KEGG_REGULATION_OF_AUTOPHAGY | ESCA, GBM, LUSC, OV | 4 |
| KEGG_RIG_I_LIKE_RECEPTOR_SIGNALING_PATHWAY | ESCA, GBM, LUSC, THYM | 4 |
| KEGG_BASE_EXCISION_REPAIR | PRAD, STAD, THYM | 3 |
| KEGG_CALCIUM_SIGNALING_PATHWAY | ESCA, STAD, THCA | 3 |
| KEGG_DRUG_METABOLISM_CYTOCHROME_P450 | BLCA, READ, STAD | 3 |
| KEGG_GRAFT_VERSUS_HOST_DISEASE | BLCA, BRCA, THCA | 3 |
| KEGG_HOMOLOGOUS_RECOMBINATION | PRAD, STAD, THYM | 3 |
| KEGG_HYPERTROPHIC_CARDIOMYOPATHY_HCM | HNSC, STAD, THCA | 3 |
| KEGG_LEISHMANIA_INFECTION | BLCA, ESCA, THCA | 3 |
| KEGG_PORPHYRIN_AND_CHLOROPHYLL_METABOLISM | BRCA, SARC, UCEC | 3 |
| KEGG_RETINOL_METABOLISM | READ, STAD, THYM | 3 |
| KEGG_SYSTEMIC_LUPUS_ERYTHEMATOSUS | BRCA, OV, THCA | 3 |
| KEGG_TOLL_LIKE_RECEPTOR_SIGNALING_PATHWAY | BLCA, GBM, LUSC | 3 |

**S1 Table. The KEGG pathway that RRM2 regulates in pan-cancer by using GSEA.**

| **Drug Target** | **Drug Name (Approved)** | **Drug ID** |
| --- | --- | --- |
| Histone deacetylase 1 (HDAC1) | Phenylbutyrate | D0B7CH |
| Histone deacetylase 1 (HDAC1) | Romidepsin | D0L7LC |
| Histone deacetylase 1 (HDAC1) | ITF2357 | D0GH3Q |
| Tyrosine-protein kinase ABL1 (ABL) | Adenosine triphosphate | D01BYB |
| Tyrosine-protein kinase ABL1 (ABL) | Bosutinib | D0OB0F |
| Tyrosine-protein kinase ABL1 (ABL) | SKI-758 | D03MNN |
| Tyrosine-protein kinase ABL1 (ABL) | Ponatinib | D0H0EQ |
| Cyclin-dependent kinase 4 (CDK4) | Apremilast | D07ESC |
| Cyclin-dependent kinase 4 (CDK4) | LY2835219 | D05SBO |
| Cyclin-dependent kinase 4 (CDK4) | Ribociclib Succinate | D01HVT |
| Cyclin-dependent kinase 6 (CDK6) | Apremilast | D07ESC |
| Cyclin-dependent kinase 6 (CDK6) | LY2835219 | D05SBO |
| Cyclin-dependent kinase 6 (CDK6) | Ribociclib Succinate | D01HVT |
| Transforming growth factor beta 1 (TGFB1) | Pirfenidone | D02WCI |

**S2 table. Potential drug prediction of RRM2 for pan-cancer**
