# Supplementary material for: A comprehensive analysis of ribonucleotide reductase subunit M2 for carcinogenesis in pan-cancer
Source: PLoS One. 2024 Apr 18;19(4):e0299949. doi: 10.1371/journal.pone.0299949 (PMC11025932; doi:10.1371/journal.pone.0299949)
Supplement: Attachment — Submitted filename: Response to Reviewers.docx [file pone.0299949.s002.docx]

**Response to Reviewers**

Reviewer #1: This study addressed the role of RRM2 in various tumors, which provide some interesting

information for understanding carcinogenesis. There are some flaws needed to be addressed, as followings.

1- English should be carefully revised throughout the manuscript.

A: I've done a native polish on the entire manuscript by Elsevier professional language polishing services.

2- The quality of figures is very poor, and should be improved.

A: All the figures have been improved.

3- Introduction, page 3, line 22: “…to perform pan-cancer analysis of genes…” should be “…to perform a pan-cancer analysis of genes…”

A: “…to perform pan-cancer analysis of genes…” has been modified into “…to perform pan-cancer analyses of genes…”according to Elsevier professional language polishing services.

4- Description of RRM2 gene expression should be in Italic and consistent through the manuscript.

A: All description of RRM2 gene expression have be revised in Italic and consistent through the manuscript.

5- Material and methods, page 4, line 78: “we chose…” should be “We chose…”

A: “we chose…” has been modified into “We chose…”

6- Material and methods, page 4, line 79: “…immune infiltrates of endothelial cell…” should be “…immune infiltrates of endothelial cells…”

A: …immune infiltrates of endothelial cell…” has been modified into “…immune infiltrates of endothelial cells…”

7- Material and methods, page 5, line 97: “[24)” should be “[24]”

A: “[24)” has been modified into “[24]”

8- Material and methods, page 5, line 97: “pan-cencer” should be “pan-cancer”

A: “pan-cencer” has been modified into “pan-cancer”

9- Material and methods, page 4, “Genetic alteration analysis section”: In the analysis of RRM2 gene mutations, it is better to provide the accession number of RRM2 coding sequence used for the analysis.

A: The number of RRM2 coding sequence is NC_000002.12NC_000002.12

10- Results, page 6, “RRM2 expression and clinical correlation in pan-cancer”: The authors examined the correlation between RRM2 expression and clinical characteristics of cancer patients, and found that RRM2 expression was significantly related to age, race, tumor stage, and status of cancer patients. It is better to describe in the main text, which age, race and tumor stage are associated with high RRM2 expression level.

A: The clinical characteristics of patients have been described in the main text, which age, race and tumor stage are associated with high RRM2 expression level.

11- Results, page 6, line 143-144: “…tumor patients’ clinical characteristics of patients with pan-cancer…” should be “…tumor clinical characteristics of patients with pan-cancer…”

A: “…tumor patients’ clinical characteristics of patients with pan-cancer…”have been modified into “…the clinical characteristics of patients with pan-cancer…”according to Elsevier professional language polishing services.

12- Results, page 9, line 218: “…tumor mutational burden) TMB) …” should be “…tumor mutational burden (TMB)…”

A: “…tumor mutational burden) TMB)…” has been modified into “…tumor mutational burden(TMB)…”

13- Results, page 10, line 273: “…in Fig 7A We the…” should be “…in Fig 7A. We the…”

A: “…in Fig 7A We the…” has been modified into “…in Fig 7A. We the…”

14- Discussion, page 13, lines 376-379: the authors found that “cell cycle”, “p53 signaling pathway”, “cell senescence, and other cellular pathways have potential roles in the effect of RRM2 on carcinogenesis. It is better to discuss these results according to the literature data.

A: The “cell cycle”, “p53 signaling pathway”, “cell senescence, and other cellular pathways have potential roles in the effect of RRM2 on carcinogenesis. I discussed these results according to the literature data.

Reviewer #2: Numerous experimental studies have been conducted to show that RRM2 is associated

with the occurrence and development of tumors. And the function and mechanism of RRM2 in

tumors have also been relatively well studied. Therefore, this manuscript lacks novelty.

A: Indeed, many studies have reported the close relationship between RRM2 and tumorigenesis and development, but most of them still focus on tumor invasion and proliferation and corresponding signaling pathways and other tumor molecular mechanisms. In this paper, we have made a relatively new interpretation of the mechanism of RRM2 regulation of tumor from the perspective of the immune mechanism of RRM2 and tumor-infiltrating lymphocytes and immune checkpoint molecules, which may provide certain theoretical support for tumor immunotherapy.
