# Supplementary material for: A comprehensive analysis of ribonucleotide reductase subunit M2 for carcinogenesis in pan-cancer
Source: PLoS One. 2024 Apr 18;19(4):e0299949. doi: 10.1371/journal.pone.0299949 (PMC11025932; doi:10.1371/journal.pone.0299949)
Supplement: Attachment — Submitted filename: Response to Reviewers.docx [file pone.0299949.s003.docx]

Response to Reviewers

Reviewer #2:

1. Why did "Enrichment analysis of SND1-related partners"? Is there a relationship of RRM2 and SND1?

A: I'm very sorry. Thank you for your careful review. This is actually a clerical error on my part. It's actually “Enrichment analysis of RRM2-related gene”. I have already corrected all the relevant clerical errors in the Methods and Results.

2. Please consider to cite "https://doi.org/10.1016/j.gendis.2022.11.022".

A: Sorry, I don't know what this link means and where to reference it. Besides, this link is typed to show AN ERROR with DOI NOT FOUND.

3. All figures have to be uploaded in high resolution.

A: All figures have been uploaded in high resolution
